# Absence of Tuberculosis-Causing *Mycobacteria* from Slaughtered Livestock Tissues and Environmental Samples, Gauteng Province, South Africa

**DOI:** 10.1155/2024/4636652

**Published:** 2024-03-15

**Authors:** Vuyokazi Mareledwane, Abiodun A. Adesiyun, Tiny M. Hlokwe

**Affiliations:** ^1^Department of Production Animal Studies, Faculty of Veterinary Science, University of Pretoria, Private Bag X04, Onderstepoort 0110, Pretoria, South Africa; ^2^Bacteriology Section, Diagnostic Services Programme, Agricultural Research Council-Onderstepoort Veterinary Institute, Private Bag X05, Onderstepoort 0110, Pretoria, South Africa; ^3^School of Veterinary Medicine, Faculty of Medical Sciences, University of the West Indies, St. Augustine, Trinidad and Tobago

## Abstract

*Mycobacterium tuberculosis* complex (MTBC) is a group of bacteria responsible for causing tuberculosis in animals and humans. In South Africa (S.A), slaughterhouses are registered by the government and closely inspected and audited for hygienic slaughter practices. Meat inspection to detect lesions has been used for passive surveillance, monitoring, and diagnosis of the disease status. Information on the current status of bovine tuberculosis (bTB) in livestock in the country is limited. Hence, we investigated the occurrence of *Mycobacterium* spp. in the tissues of slaughtered livestock and environmental samples in abattoirs in Gauteng province of South Africa (S.A). The cross-sectional study employing random sampling from cattle, pigs, and sheep (with the collection of liver, lung, spleen, and different lymph nodes) irrespective of lesions was carried out in 19 red meat abattoirs. Five hundred animals were sampled, comprising cattle (*n* = 369), pigs (*n* = 90), and sheep (*n* = 41). Additionally, 19 environmental samples were collected from feedlots, or where animals drink water while awaiting slaughter, to identify mycobacterial species using culture, acid-fast bacteria staining, and polymerase chain reaction (PCR). The Chi-square and Fisher's Exact tests were used to detect statistically significant differences in the frequency of detection of *Mycobacterium* spp. according to the variables investigated (types of tissues, livestock, abattoirs, etc.). The PCR assays detected no MTBC complex species DNA in the bacterial isolates from cattle (*n* = 32). Sequence analysis (16S rDNA) of the isolates from eight cattle confirmed only two species, namely *Mycobacterium colombiense* (99.81% identity) and *Mycobacterium simiae* (99.42% identity). The remaining isolates were identified as members of the *Actinomadura* species. From the environmental samples, bacterial isolation was made from three samples, and two could only be identified up to the genus level (*Mycobacterium* species) while the remaining isolate was identified as *Mycobacterium senuense* (99.22% identity). The study revealed the absence of bovine tuberculosis-causing pathogens in red meat abattoirs of the Gauteng province. Although non-tuberculous *Mycobacteria* have been implicated as potentially causing tuberculosis-like diseases in livestock, their occurrence in the current study was found to be low, but the potential to cause disease cannot be ignored.

## 1. Introduction

Bovine tuberculosis (bTB), caused mainly by *Mycobacterium bovis*, is a zoonotic disease with economic losses estimated at billions of dollars annually worldwide, yet the economic impact of the disease in African countries remains unquantified [1]. In South Africa, TB in cattle is mostly known to be caused by *M. bovis* and it is classified as a controlled disease according to the national Animal Diseases Act, Act 35 of 1984. Control strategies have been put in place since 1969, and reduced the disease prevalence in communal cattle herds to less than 1%, although sporadic outbreaks still occur [2, 3]. The true disease prevalence in different provinces of the country including Gauteng province is however, currently unknown, more especially in communal cattle populations. This is mostly due to the fact that control of the disease has become less prioritised by the government over the years due to lack of funding [4].

In S.A, slaughtering of livestock is officially conducted at abattoirs, although some back yard slaughtering also occur. Official abattoirs are registered by government and closely inspected and audited for hygienic slaughter practices [4]. Meat inspection in slaughterhouses to detect lesions has been used for passive surveillance, monitoring, and diagnosis of the disease [5]. However, this method only detects lesions in advanced stages of infection when visible lesions are observed [6]. Furthermore, it should be noted that lesions observed are not only due to *M. bovis* or other members of the *Mycobacterium tuberculosis* complex (MTBC), but some members of the *Mycobacterium avium* complex (MAC) and other non-tuberculous mycobacteria (NTM) are also both pathogenic and opportunistic and may produce lesions in the infected animals [7]. Hence, confirmation of the causative agent is of outmost importance, as NTM were previously also found to interfere with cell mediated immunity (CMI) response diagnostic tests for bTB [8, 9]. Studies have suggested that meat inspection should be done together with routine culturing and followed by molecular methods for characterizing different mycobacterial species [10] especially in regions with a high TB burden and where NTMs remain undiagnosed [11]. It has been reported that approximately one-third of the NTMs are responsible for human disease [12]. In the last three decades, there has been a recorded increase in NTM laboratory isolation [13]. In a study by Oloya [14] in Uganda, 19 *M. bovis* and 11 NTM were isolated from 61 tissue samples from slaughter livestock, confirming the widespread prevalence of bTB in this country. In another study, Awah-Ndukum et al. [15] confirmed the presence of bTB by isolating *M. bovis* from samples collected at abattoirs. In a recent study, from Rwanda, both *M. bovis* and *Mycobacterium tuberculosis* were isolated from slaughtered cattle, indicating that bTB is present in this country, although at low prevalence [16]. Molecular epidemiology of bTB is useful in the determination of risk factors of bTB transmissions, identification of the sources of contamination, tracking of the geographic distribution, and the spread of Mycobacterial species.

Information on the current status of bTB in livestock in the country is limited. Hence, we investigated the occurrence of *Mycobacterium* spp. in the tissues of slaughtered livestock and environmental samples in abattoirs in Gauteng province of S.A and determined the possible food safety risks posed to meat consumers.

## 2. Materials and Methods

### 2.1. Description of the Study Area, Abattoirs, Sample Type, and Size Determination

**Figure 1:**
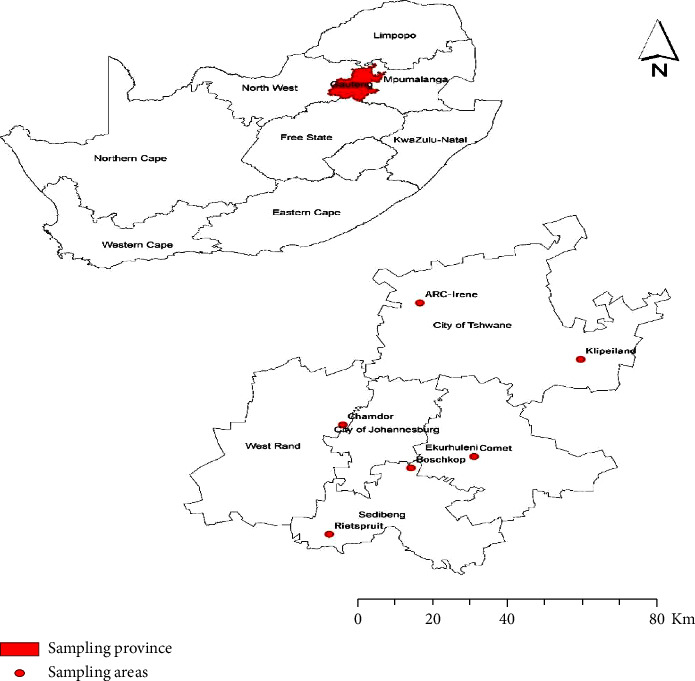
Map showing the six districts where red meat abattoirs sampled are located within the Gauteng province of South Africa.

This study was conducted in 19 red meat abattoirs across the Gauteng province of South Africa and was done in parallel with our previous study [17]. Briefly, the investigators obtained a list of functional red meat abattoirs (mono- and multi-species) located across Gauteng province from the Gauteng Department of Agriculture and Rural Development (GDARD). Based on the information provided for each abattoir which included the type and number of livestock slaughtered daily, location, and their facilities, a total of 19 abattoirs were randomly selected, comprising 16 high-throughput (HT) and three low-throughput (LT) abattoirs for the study. The study was conducted in all six districts of Gauteng province, namely, the City of Johannesburg, the City of Tshwane, Ekurhuleni, Metsweding, Sedibeng, and West Rand (Figure 1). At each selected abattoir, slaughtered cattle, pigs, and sheep were sampled on a single day using a systematic random sampling method, and the abattoir setting determined this. Approximately 30 animals were sampled during each sampling visit to the abattoir, and the number sampled per livestock species was proportional to the expected number to be slaughtered on that day, as provided by the abattoir workers. The prevalence of *Mycobacterium* spp. in tissues collected from slaughtered livestock in South Africa is currently unknown. The prevalence was therefore estimated to be 50% based on the standard practice used for unknown prevalence, and sample size determination was calculated using the formula *n*_*o*_ = 1.96^2^ × *P*_exp_ × (1 − *P*_exp_)/*d*^2^ recommended by Thrusfield [18], where *n*_*o*_ represented the minimum sample size, *P*_exp_ represented the expected prevalence and *d*^2^ represented the desired precision value of 5%. Although the minimum estimated sample size was 384, a total of 500 animals were sampled, and the distribution per livestock species (cattle, sheep, and pigs) was proportional to the number of animals slaughtered per species during abattoir visits.

### 2.2. Pre- and Post-Slaughter Inspection

The professional meat inspectors assigned to each abattoir conducted pre- and post-slaughter inspections according to a standard procedure, which included detecting clinical TB lesions as previously described [17]. Biographical information about the slaughtered livestock, including gender, breed, district, and municipality origin of animals, was recorded [17].

### 2.3. Sample Collection

While the inspectors were conducting physical examinations of the carcasses, the following organs were collected, i.e., lymph nodes (retropharyngeal, abdominal, mesenteric), liver, lung, and spleen for the *Mycobacterium* species culture test. A total of 500 animals were sampled, i.e., cattle (*n* = 369), pigs (*n* = 90), and sheep (*n* = 41). Overall, 2000 tissue samples were collected from different organs of each animal. Tissue samples were labelled and placed in sterile Ziploc bags or specimen containers. All the tissues were transported to the Tuberculosis laboratory at the Agricultural Research Council-Onderstepoort Veterinary Research (ARC-OVR) in ice-cooled boxes. Environmental samples (water; *n* = 19), one from each abattoir, were collected from feedlots or where animals drink water while waiting for slaughter. All the collected environmental and tissue samples were cultured according to established standard laboratory protocols and monitored weekly for bacterial growth.

### 2.4. Isolation of *Mycobacterium* Species from Tissue Samples

To culture tissue samples for *Mycobacterium* spp., standard methods available in the ARC-OVR Tuberculosis laboratory were used [8]. Approximately 944 samples from 236 animals were individually processed. Representatives from the same animals were processed as a pool for the remaining 1056 samples (264). Briefly, the collected tissue samples were cut into approximately 5 g pieces, and all the fat was removed. Using the Ultra-Turrax® homogenizer (Separation Scientific, SA), the samples were then homogenized. The homogenates were poured into two 50 ml tubes in preparation for the decontamination. Approximately 7 ml of each homogenized sample was transferred into two 15 ml centrifuge tubes each and then decontaminated in the first tube with HCL to a final concentration of 1.0% and in the second tube with NaOH to a final concentration of 2.0% for 10 minutes, followed by centrifugation (3500 rpm). The Lowenstein-Jensen (LJ) media supplemented with pyruvate (4 slopes) and glycerol (2 slopes) was inoculated with the sample pellets following neutralization with sterile distilled water and centrifugation (3500 rpm). For each batch of samples tested, positive and negative controls were included. The positive control was a sample collected from either skin test positive/suspect reactor or slaughter cattle with suspect tuberculous lesions and confirmed by culture and PCR, while the negative control sample was collected from a carcass declared fit for human consumption and underwent microbiological testing for confirmation. The inoculated media slopes were incubated at 37°C for 10 weeks and examined weekly for bacterial growth (colonies).

### 2.5. Isolation of *Mycobacterium* Species from Water Samples

For the isolation of *Mycobacterium* species, water samples were collected from the selected abattoirs and cultured according to the standard laboratory protocol at the Tuberculosis laboratory. Positive (water sample spiked with a colony of an in-house *Mycobacterium bovis*, TB 9854A) and negative (unspiked sterile distilled water) controls were included for each batch of samples tested. Briefly, 2% NaOH was added to the samples and centrifuged, thereafter 5.0% oxalic acid was added. After centrifugation, Lowenstein-Jensen (LJ) slopes (3x) were inoculated with the sample and incubated at 27°C. Also, another set was inoculated and incubated at 37°C and monitored for up to 10 weeks for colonies typical of *Mycobacterium* species. Thereafter, colonies were selected for Ziehl-Neelsen staining and PCR identification.

### 2.6. Ziehl-Neelsen Staining and Microscopy

The presence of colonies following the detection of growth on the selective media after incubation was suggestive of the presence of *Mycobacteria*, and bacterial smears were prepared on microscopic slides for Ziehl-Neelsen (ZN) staining to confirm acid-fastness. The ZN-stained smears were then observed under a microscope.

### 2.7. DNA Extraction in Preparation for Polymerase Chain Reaction

DNA extraction was conducted as previously described [19]. DNA templates were prepared from colonies typical of *Mycobacterium* species, and individual colonies were picked up from the L-J media and mixed with 100 *μ*l ultra-pure water. DNA was extracted from isolates by heat-treating the suspension at 100°C for 25 minutes and allowed to cool down at room temperature. DNA templates were stored at −20°C until PCR analysis [19].

### 2.8. Identification of *Mycobacterium tuberculosis* Complex Species

To identify *Mycobacterium tuberculosis* complex species (MTBC), a modified PCR assay was conducted using primers targeting the region encoding MPB 70 antigen belonging to MTBC (forward primer: 5′ GAACAATCCGGAGTTGACAA 3′ and reverse primer: 5′ AGCACGCTGTCAATCATGTA 3′) [20]. Briefly: a 50 *μ*l reaction consisting of 25 *μ*l ultra-pure water, 5 *μ* of 10x buffer, 3 *μ*l of 25 mM MgCl_2_, 2.5 *μ*l of dNTP mix (1 mM), 2 *μ*l of each TB 1A (20 pmol/*μ*l) and TB 1B (20 pmol/*μ*l) primers, 10 *μ*l of the acid-fast bacterial lysate (DNA template). The enzyme 0.5 *μ*l of Taq polymerase (supertherm) was added, and the reaction mixture was placed in a thermocycler under appropriate PCR conditions. PCR cycling conditions were as follows: Initial denaturation at 94°C for 5 minutes, denaturation at 94°C for 30 seconds, annealing at 64°C for 30 seconds, and extension at 72°C for 2 minutes for 40 cycles [19, 20].

### 2.9. Gel Electrophoresis

The PCR products were visualized on a 1.5% agarose gel stained with 20 *μ*l ethidium bromide (10 *μ*g/ml) and run at 80 V for 3 h. A 100 bp ladder (Inqaba Biotechnical Industries) was included and used to estimate the size of the resulting PCR products. Positive and negative controls (previously tested DNA templates) were included for quality control. In addition, distilled water was included as a blank control.

### 2.10. Identification of Non-Tuberculous *Mycobacterium* spp. by PCR and Sequence Analysis

Non-tuberculous mycobacteria (NTM) were identified by PCR and sequence analysis of the 577 bp of the *Mycobacterium* 16S rDNA gene using the following primers: 16S rDNA forward 5′ AGA GTT TGA TCC TGG CTC AG 3′ and 16S rRNA reverse 5′ GCG ACA AAC CAC CTA CGA G 3′ as previously described [9]. The mycobacterial cell lysate was used as a DNA template in a 25 *μ*l PCR mixture containing 12.4 *μ*l deionized water, 2.5 *μ*l of 10x PCR buffer (160 mM) (Tris Cl, KCl, (NH_4_)_2_SO4), 2 *μ*l MgCl_2_ (25 mM), 1 *μ*l dNTPs (10 mM), 0.1 *μ*l Taq polymerase (Qiagen Hotstar Taq, Whitehead Scientific, South Africa), 5 *μ*l of 5x Q-solution, 1 *μ*l of each forward and reverse primers (50 pmol) and 1-2 *μ*l DNA template. The PCR cycling parameters were as follows: initial denaturation at 95°C for 15 minutes, followed by 35 cycles of denaturation at 95°C for 30 seconds, annealing at 60°C for 30 seconds, and elongation at 72°C for 30 seconds and a final extension at 72°C for 10 minutes. The amplicons were sent to Inqaba Biotechnical Industries, Ltd, SA, for the forward 16S rRNA gene sequencing using an ABI sequencer. Sequences were edited manually, and pairwise alignments were undertaken using the BioEdit Sequence alignment editor (version 7.1.9). The sequences were analyzed on the NCBI BLAST platform for species identification by the mega blast [9].

### 2.11. Data Analysis

The data obtained was entered into Microsoft Excel (Microsoft, United States) database and only descriptive analysis was conducted. Analysis of the data was based on the prevalence of *Mycobacterium* spp. based on individual positivity, regardless of the tissues (lymph nodes, liver, lungs, and spleen). For the study, the dependent variable was the isolation of *Mycobacterium* spp., and the independent variables were the type, gender, and breed of animal species, livestock origin, district, municipality, and abattoirs (HT and LT). Chi-square and Fisher's Exact analysis were conducted to determine whether there were statistically significant differences in the frequency of isolation of *Mycobacterium* spp. among the livestock tissues tested according to the independent variables mentioned above. The level of significance was determined at an alpha level of 0.05.

## 3. Results

### 3.1. Sample Collection

A total of 500 animals were sampled, comprising 369 adult cattle (Bonsmara, *n* = 277; Jersey, *n* = 39; Nguni, *n* = 51; Brahman, *n* = 1; Holstein, *n* = 1), 90 pigs and 41 sheep (Dorper) of any gender were collected (Tables 1–3).

### 3.2. Bacterial Isolation from Tissue Samples and Ziehl Neelsen Staining

Tissue samples originating from 19 different red meat abattoirs located in the Gauteng province (Figure 1) were collected and tested at the Tuberculosis Laboratory at the ARC-OVR for *Mycobacterium* spp. isolation. Colonies of bacterial growth typical of *mycobacteria* were observed. Bacterial isolation was made from 32 samples representing individual cattle from 12 different red meat abattoirs. Because of the single colony growth from most of the samples, only a few could be subjected to Ziehl Neelsen staining to confirm acid fastness. No isolation was made from pig and sheep samples.

### 3.3. Identification of *Mycobacterium tuberculosis* Complex Species by PCR

Out of the 32 isolates, no MTBC complex species was detected.

### 3.4. Characterization of *Mycobacterium* Isolates from Tissue Samples by 16S rRNA PCR and Gene Sequence Analysis

**Figure 2:**
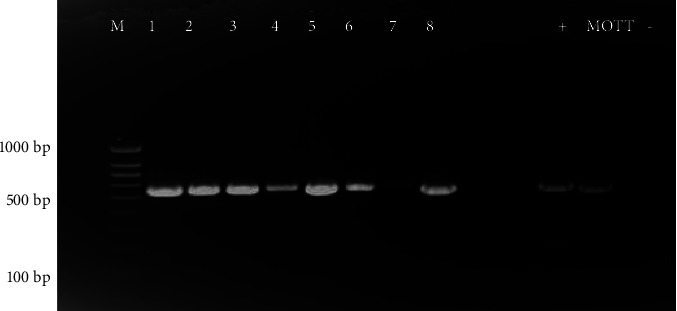
Gel electrophoresis results obtained from 16S rRNA PCR analysis. Lane M is a 100 bp DNA marker; lane 1 represents an isolate from abattoir N, lanes 2–4 represent isolates from abattoir P, lanes 5–7 represent isolates from abattoir B while lane 8 represents an isolate from abattoir K, lane + represent positive control (TB 9845A), lane MOTT represents a *Mycobacteria* positive control (TB9801B) and lane-represents the negative control which was distilled water.

Out of the 32 isolates, 8 (25%) from individual animals originating from 4 abattoirs displayed a 577 bp PCR product by 16S rRNA PCR analysis (Figure 2). The 16S rRNA gene sequence analysis following NCBI blasting identified only two of the isolates as *Mycobacteria*, i.e., *Mycobacterium colombiense* (99.81% identity) isolated from cattle from animal # 18, which originated from abattoir P; and *Mycobacterium simiae* (99.42%) isolated from cattle from animal # 27 sampled at abattoir K. The remaining 6 isolates were identified as members of the *Actinomadura* species, hence the overall prevalence of *Mycobacterium* spp. in cattle slaughtered at Gauteng abattoirs was estimated to be 0.5%.

### 3.5. Isolation of *Mycobacteria* from Environmental Samples

Bacterial isolation was made from water samples from five abattoirs: L, P, O, G, and D. All isolates were confirmed as acid-fast by Ziehl-Neelsen staining.

### 3.6. Characterization of *Mycobacterium* spp. from Environmental Samples by 16S rRNA PCR and Gene Sequence Analysis

Bacterial isolation was made from five of the 19 (26.3%) environmental samples. Specific PCR amplification was obtained from four of the five (15.8%) acid-fast isolates, and the remaining isolate was negative. The expected size of the PCR product was approximately 577 bp indicative of a mycobacterium species or closely related species. A weak PCR product was however observed for an isolate from abattoir P (results not shown), hence it could not be submitted for sequencing. Sequence data analysis following NCBI blasting identified one of the isolates up to genus level (*Mycobacterium* species at 98.89% identity). This isolate was from a sample collected at the abattoir G. *Mycobacterium avium* (99.61% identity) was isolated from a sample collected at abattoir L, and *Mycobacterium senuense* (99.02% identity) from abattoir D. The overall prevalence of *Mycobacteria* isolated from environmental samples was 15.8%.

## 4. Discussion

In this study, no MTBC species were isolated from tissues of slaughtered livestock and environmental samples in abattoirs in the Gauteng province of S.A, and no possible food safety risks were posed to meat consumers. This is not a surprise because no lesions resembling *M. bovis* infection were visible on any of the samples processed. Tuberculosis-like lesions are an indicator of the presence of tuberculosis [21, 22]. According to Botha et al. [23], tuberculosis is a slow and progressive disease that remains asymptomatic for an extended period until the advanced stages of infection when lesions appear. This may have been the case in this study that when livestock are taken to the abattoirs for slaughter, the animals could still be in the early stages of infection and the bacterial concentrations are too low to be detected by culture. In South Africa, implementing the national bovine tuberculosis programme, especially in commercial cattle, decreased the national prevalence of bovine tuberculosis to 0.4% over 25 years ago, from 11.85% over half-century ago [3] with sporadic outbreaks still occurring [2]. In communal cattle farming, the disease prevalence is largely still unknown, with a few isolated studies reporting varying prevalence ranging from less than 0.5% to more than 15% [24, 25]. It should be noted that animals sampled in the current study were from auctions and feedlots originating from different provinces of the country. The prevalence of bovine tuberculosis in SA is however, still low contrary to other African countries such as Zambia, Uganda, and Ethiopia, where herd prevalence of up to 50% have been reported in cattle [14, 26, 27]. No isolation was made from pigs and sheep sampled in the current study. Tuberculosis in sheep is generally rare and there are conflicting opinions regarding the susceptibility of sheep to MTBC species. Considering the chronic nature of the disease, potentially infected sheep get slaughtered before the disease develops [28, 29]. With that said, a few studies have however, reported cases of *M. bovis* infection in sheep [29–31]. Although bovine tuberculosis in sheep has never been reported in South Africa [17], known zoonotic mycobacteria, including *Mycobacterium avium* subsp. *hominisuis*, *Mycobacterium avium* subsp. *avium*, *Mycobacterium intracellulare*, *Mycobacterium bovis* and *Mycobacterium tuberculosis* were previously isolated from slaughtered pigs during the years 1991–2002, hence a potential public health concern [32].

In our previous study, conducted in parallel with the current research, Mareledwane [17] reported an estimated bTB seroprevalence of 4.4% in cattle, indicating past exposure or detection of early infection, an advantage of the cell-mediated immune response-based tests. Although a 0.0% prevalence (absence) for MTBC species was recorded in the current study, NTM were isolated, with the overall prevalence of *Mycobacterium* spp. estimated to be 0.5% for cattle and 15.8% for environmental sample. Non-tuberculous mycobacteria isolated include *Mycobacterium colombiense*, *Mycobacterium simiae*, *Mycobacterium avium*, and *Mycobacterium senuense*. It is well known that non-tuberculous mycobacteria interfere with the most widely used tests for the diagnosis of bovine tuberculosis such as the intradermal tuberculin test as well as the gamma interferon test, hence of outmost importance to identify such interferences [9, 33]. The NTMs identified in the current study were previously found to occur in the environment and animals during a country wide study conducted [9].

One of the NTM isolated in our study, *M. simiae*, has been shown to cause pathological conditions, especially in humans, and has been isolated more especially in immunocompromised patients [34]. It has been reported that infection by this NTM leads to extrapulmonary infections [35].

This is the first study to investigate the occurrence of *Mycobacteria* and to isolate NTMs from slaughtered cattle in red meat abattoirs in the Gauteng province of SA. In a previous report, Hlokwe and co-workers isolated *Mycobacterium nonchromogenicum* and an NTM species closely related to *Mycobacterium moriokaense* from cattle on two farms in the Eastern Cape province of South Africa. Similar to the outcome of the current study, these NTM species were detected from samples without visible lesions, hence their clinical significance could not be determined but the potential to cause disease cannot be ignored [8].

## 5. Conclusion

The current study demonstrated a very low prevalence of *Mycobacterium* species in Gauteng red meat abattoirs as confirmed by culture and molecular assays, hence posing a low risk of infection to meat consumers. Importantly, the study demonstrated the absence (0.0% prevalence) of MTBC species in all the abattoir samples tested. However, future studies should pay attention to NTM isolated from abattoirs, as their role in causing mycobacteriosis both in humans and animals still needs to be better understood.

## Figures and Tables

**Table 1:** Characteristics of cattle sampled and the estimated prevalence of *Mycobacterium* species in Gauteng abattoirs.

Variable	Level	*N*	Prevalence (%)
Species	Bovine	369	0.54

Gender	Female	92	0
	Male	277	0.54

Breed	Bonsmara	275	0.54
	Nguni	51	0
	Jersey	43	0
	Brahman	0	0
	Holstein	0	0

District	Tshwane	156	0.27
	Sedibeng	112	0
	Metsweding	14	0
	West Rand	30	0
	Ekurhuleni	57	0.27

Municipality	City of Tshwane	156	0.27
	Ekurhuleni Metro	57	0.27
	Emfuleni	52	0
	Kungwini	14	0
	Lesedi	60	0
	Mogale City	30	0

Abattoirs	Abattoir A	30	0
	Abattoir B	30	0
	Abattoir C	30	0
	Abattoir D	22	0
	Abattoir E	30	0
	Abattoir P	27	0.27
	Abattoir G	30	0
	Abattoir H	30	0
	Abattoir I	30	0
	Abattoir J	30	0
	Abattoir K	28	0.27
	Abattoir M	30	0
	Abattoir N	14	0
	Abattoir O	8	0

Origin of animals	Auctions	58	0.27
	Farm/feedlots	311	0.27

Abattoir type	HT-multi	347	0.54
	LT-multi	22	0

Total		369	0.54

*N*: indicates number of cattle sampled.

**Table 2:** Characteristics of sheep sampled in the study and the estimated prevalence of *Mycobacterium* species in Gauteng abattoirs.

Variable	Level	*N*	Prevalence (%)
Species	Ovine	41	0

Gender	Female	14	0
	Male	27	0

Breed	Dorper	41	0

District	Tshwane	34	0
	Metsweding	7	0

Municipality	City of Tshwane	4	0
	Kungwini	37	0

Abattoir	Abattoir L	30	0
	Abattoir N	7	0
	Abattoir K	4	0

Origin of animals	Auctions	4	0
	Farm/feedlots	37	0

Abattoir type	HT-multi	34	0
	LT-multi	7	0

Total		41	0

*N*: indicates the number of sheep sampled.

**Table 3:** Characteristics of pigs sampled in the study and the estimated prevalence of *Mycobacterium* species in Gauteng abattoirs.

Variable	Level	*N*	Prevalence (%)
Species	Porcine	90	0

Gender	Female	40	0
	Male	50	0

Breed	Large white	90	0

District	Sedibeng	50	0
	West Rand	20	0
	Ekurhuleni	20	0

Municipality	City of Johannesburg	20	0
	Ekurhuleni Metro	20	0
	Midvaal	20	0
	Lesedi	30	0

Abattoirs	Abattoir F	30	0
	Abattoir Q	20	0
	Abattoir R	20	0
	Abattoir S	20	0

Origin of animals	Auctions	30	0
	Farm/feedlots	60	0

Abattoir type	HT-multi	50	0
	LT-multi	40	0

Total		90	0

*N*: indicates the number of pigs samples.

## Data Availability

All data are available from the author upon reasonable request.
